# A rare case of atypical AVNRT in a patient with Ebstein anomaly

**DOI:** 10.1016/j.hroo.2024.08.005

**Published:** 2024-10-21

**Authors:** Sai Vikram Alampoondi Venkataramanan, Robert Schneider, Shane Tsai

**Affiliations:** Division of Cardiology, Department of Internal Medicine, University of Nebraska Medical Center, Omaha, Nebraska

**Key Findings**

▪ Even though accessory pathway–mediated atrioventricular re-entrant tachycardia is the commonest cause of supraventricular tachycardia (SVT) in Ebstein anomaly, atypical atrioventricular nodal re-entrant tachycardia is a rare cause of SVT in these patients and should be considered in the differential.
▪ While a ventriculoatrial linking maneuver can differentiate between atrioventricular nodal–dependent and atrioventricular nodal–independent SVTs, additional maneuvers such as ventricular overdrive pacing can help differentiate between various nodal-dependent SVTs.
▪ During electrophysiology study, it is important to be cognizant of the anatomical changes caused by the apical displacement of septal tricuspid leaflet in Ebstein anomaly.

## Introduction

Accessory pathway (AP)–mediated atrioventricular re-entrant tachycardia (AVRT) is the most common form of supraventricular tachycardia (SVT) in Ebstein anomaly of the tricuspid valve (ETV). Atypical atrioventricular nodal re-entrant tachycardia (AVNRT) is a rare cause (<2%) of SVT in this patient population.^1^ We present a rare case atypical AVNRT in ETV that was confirmed by electrophysiology study (EPS) and successfully treated with radiofrequency ablation.

## Patient presentation

A 35-year-old female patient with unrepaired ETV was evaluated for palpitations associated with dyspnea and lightheadedness. These episodes occurred twice a month, lasting several hours with heart rates in the 160s to 170s. She had undergone successful radiofrequency ablation of a posterior septal and a posterolateral AP 20 years earlier for similar symptoms. On examination, her blood pressure was 109/65 mm Hg, her heart rate was 77 beats/min, her respiratory rate was 16 breaths/min, and her saturation was 98% on room air. Cardiac auscultation revealed a normal S1 and S2 with a 3/6 pansystolic murmur at the left lower sternal border that was worse with inspiration. The rest of the examination was unremarkable.

## Initial workup

Electrocardiogram showed sinus rhythm with incomplete right bundle branch block. Echocardiography showed normal left ventricular size and function, severe right-sided chamber enlargement, and atrialization of right ventricle (RV) with moderate tricuspid regurgitation. The patient was asymptomatic while wearing 2-week cardiac monitor, which did not capture any arrythmias.

## Diagnosis and management

**Figure 1:**
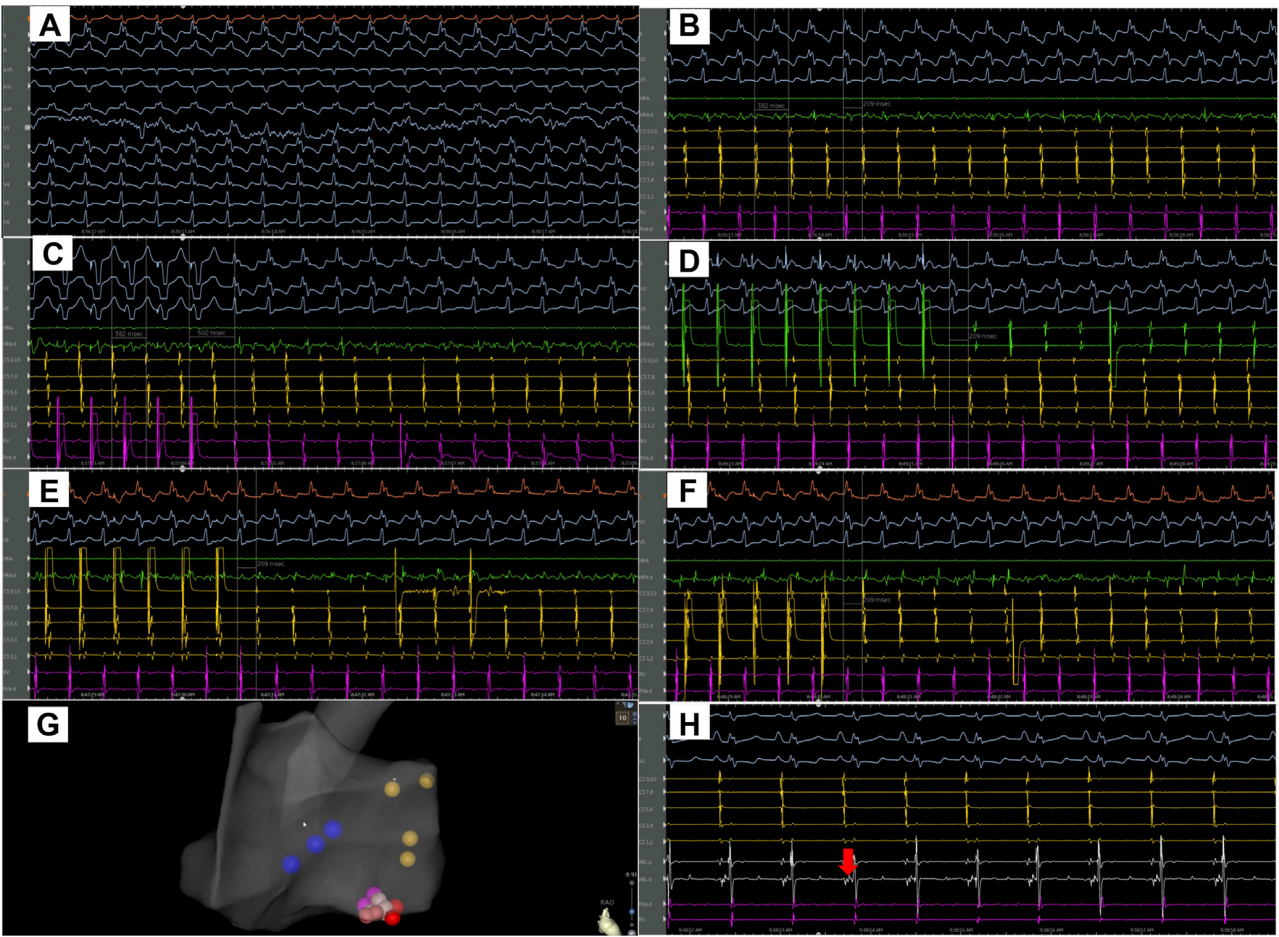
(A) Twelve-lead electrocardiography of the tachycardia. (B) Intracardiac electrogram during the tachycardia. (C) Ventricular overdrive pacing. (D–F) Differential pacing from the high right atrium, proximal coronary sinus, and distal coronary sinus shows a constant ventriculoatrial interval. (G) Right anterior oblique view of the fast anatomical map showing the posteriorly located coronary sinus (blue dots), His cloud (yellow dots), and ablation lesion (red dots). (H) Slow pathway potential (red arrow).

Because of the incessant symptoms and history of AVRT, an EPS was performed. A 5-F fixed quadripolar catheter, a 6-F quadripolar deflectable catheter, and a 7-F DecaNav F-curve decapolar catheter (Biosense Webster) were advanced to the RV apex, His bundle, and coronary sinus (CS), respectively. After marking the position of the His bundle, the 6-F quadripolar catheter was advanced to the high right atrium (RA). With ventricular pacing, there was concentric retrograde ventriculoatrial (VA) conduction, which decremented with ventricular extrastimulus pacing (ESP) down to the VA effective refractory period. With atrial ESP, a regular narrow-complex long RP tachycardia was induced (Figure 1A) with a tachycardia cycle length of 380 ms, VA interval of 210 ms (Figure 1B), and concentric atrial activation. With ventricular overdrive pacing, there was a ventriculo-atrial-ventricular response (Figure 1C), suggesting involvement of the AV node in the tachycardia circuit and excluding atrial tachycardia. The difference between the tachycardia cycle length and postpacing interval was 120 ms, which excluded AVRT, as values >115 ms are suggestive of AVNRT. With differential atrial pacing from high RA (Figure 1D), proximal CS (Figure 1E), and distal CS (Figure 1F), there was VA linking present with a constant VA interval of 210 ms. As a VA interval >70 ms is suggestive of atypical AVNRT, the patient was deemed to have atypical AVNRT. The tachycardia was terminated with ventricular burst pacing. A 4-mm nonirrigated FJ curve bidirectional ablation catheter was then advanced to the RA. Three-dimensional FAM (Fast Anatomical Mapping) of the RA was performed and integrated with the sound map (Figure 1G). The ablation catheter was positioned anterior to the ostium of the CS and directed inferiorly until the distal electrodes were recorded a small atrial deflection and a large ventricular deflection (A/V ratio 1:3 to 1:10). This suggested that the catheter was close to the tricuspid annulus. With clockwise torque of the catheter, there was significant fractioned signal noted, suggestive of a slow pathway potential (Figure 1H). Ablation was initiated inferiorly to prevent injury to fast pathway of the AV node. Radiofrequency current was applied with initial low power (10 W) and titrated upward to 45 W, with a target temperature of 50 °C. Junctional beats/rhythm were induced with ablation, and the slow pathway potential disappeared. Following ablation, extensive atrial ESP was performed without induction of tachycardia, confirming success.

## Discussion

AVRT occurs in 10% to 30% of patients with ETV.^2^ AVRT is related to the development of predominantly right-sided AP caused by apical displacement of the tricuspid valve resulting in discontinuity between the common fibrous body and the AV ring.^3^ AVNRT is less common (8%–13% of all arrythmias).^1^ AVNRT could be related to the inferior extension of the AV node, providing a substrate for slow pathway, creation of a favorable substrate from prior ablation,^4^ or fibrosis from right-sided pressure/volume overload.^4^

Catheter ablation is highly effective for curing these SVTs.^5^ During EPS, it is important to be cognizant of the anatomical changes in ETV. In patients with ETV, the anterior border of the triangle of Koch is formed by a fibromuscular ridge that separates the embryological RA from the RV and not by the septal leaflet. The CS ostium is large due to RA enlargement, and the body of the AV node is shifted inferiorly and posteriorly.^3^ Identifying these structures is critical for a successful EPS and ablation. Intracardiac echocardiography can differentiate the morphological RA and RV, while electrograms can help differentiate between the atrialized RV and RA.

Various maneuvers can help differentiate between AVRT, AVNRT, and atrial tachycardia during an EPS. In addition to ventricular overdrive pacing and VA linking maneuvers used in our case, para-Hisian pacing and adenosine infusion with V-pacing or measuring the V-stim to A interval minus VA interval could help further distinguish AVRT from atypical AVNRT. Ablation should be initiated at lower energy level and titrated slowly to avoid injury to the fast pathway, which could result in complete heart block.
